# Evolution of a Primate Defense against Intragenomic Infiltrators

**DOI:** 10.1371/journal.pbio.0020292

**Published:** 2004-07-20

Anyone who uses a word processor is likely thankful for the spell checker program. But that autocorrect function can introduce errors, “correcting” the spelling of words to fit its stored repertoire, which is decidedly limited. Take that one step further and imagine a rogue program that destroys the coherence and meaning of your prose by swapping out one letter for another throughout the document. That's the situation retroviruses like the human immunodeficiency virus (HIV) face during the course of their infectious cycle, when a protein encoded by the host genome slips into the virus, mutates the virus's genetic material, and alters the viral genome.

The gene, *APOBEC3G*, belongs to a family of primate genes that produce enzymes (in this case, APOBEC3G) that “edit” DNA and RNA, by slipping into viral particles and inducing mutations that replace one base (cytosine) with another (uracil) as the virus undergoes reverse transcription in the host cell's cytoplasm. The edited virus fails to replicate. HIV, in turn, generates a protein called Vif that binds to the APOBEC3G enzyme and targets it for degradation, thereby eliminating its antiviral activity.

Since the protein-binding regions that govern these interactions have a direct effect on the fitness of both virus and host, one would expect to see the proteins angling for advantage, with Vif maximizing its ability to recognize APOBEC3G and APOBEC3G doing its best to evade Vif. Such battles are thought to result in frequent mutations that alter the amino acids involved in the interaction; the perpetuation of such advantageous mutations is called positive selection.

In this issue of *PLoS Biology*, Sara Sawyer, Michael Emerman, and Harmit Malik investigate the genetic roots of this battle for evolutionary advantage and find something surprising. As predicted, the *APOBEC3G* gene is under strong positive selection. But that selection appears to predate the existence of HIV-type viruses.

To characterize the selective pressures on *APOBEC3G* evolution, Sawyer et al. analyzed the gene from twelve primates—New World monkeys, Old World monkeys, and great apes, including humans—spanning 33 million years of evolution. Most of the primate lineages showed evidence of positive selection, indicating that the gene has been under pressure to adapt throughout the history of primate evolution. But viruses like HIV have been found in only five of the primates studied—three African monkeys, chimpanzees, and humans—and appear to be at most one million years old. And HIV infection in human populations is too recent to account for the positive selection of *APOBEC3G* in humans—so what has been fueling *APOBEC3G'*s rapid evolution?[Fig pbio-0020292-g001]

**Figure pbio-0020292-g001:**
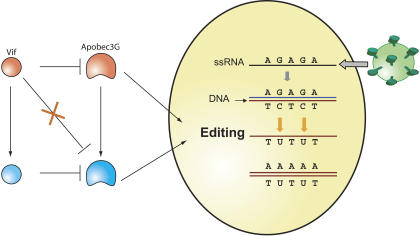
Genetic conflict between the host antiviral editing enzyme APOBEC3G, and the viral Vif protein leads to rapid fixation of amino acid replacements in both proteins

APOBEC3G and Vif interact in T-cells, but the fact that selective pressure on *APOBEC3G* has been constant over the course of primate evolution suggests that another force is also acting on the gene. Sawyer et al. propose that this force is most likely occurring in germline cells (sperm and egg precursors), which also produce high levels of *APOBEC3G* and can pass mobile genetic elements on to the next generation. Despite being non-infectious, these elements increase their own copy number in the host genome, moving from one part of the genome to another. The human genome is littered with such “retrotransposons,” and it is these mobile genetic elements, the authors conclude, that likely antagonize *APOBEC3G*.

One class of retrotransposons, called human endogenous retroviruses, acts in many ways like foreign retroviruses. A retrovirus emanating from one's own genome poses less of an immediate threat than a retrovirus like HIV. But the constant efforts of the endogenous retrovirus to “jockey for evolutionary dominance,” the authors conclude, could eventually take a toll and would be expected to provoke efforts to contain it. And it may be that this ancient intragenomic conflict endowed APOBEC3G with the means to do battle with foreign retroviruses like HIV.

Sawyer et al. also found evidence that five other *APOBEC* human genes appear to be engaged in similar conflicts. Combined with the finding that rodents have only one *APOBEC3G* gene and that five out of the six human *APOBEC3* genes have been under positive selection, these results suggest that this gene family expanded in mammalian evolution as a means of defending the germline from the promiscuous intrusions of mobile genetic elements.

